# Acute mild cold exposure with shivering reduces 24 h glucose levels in individuals with type 2 diabetes but not prediabetes

**DOI:** 10.1007/s00125-026-06809-z

**Published:** 2026-07-20

**Authors:** Dzhansel Hashim, Johanna A. Jörgensen, Tineke van de Weijer, Denis P. Blondin, Patrick Schrauwen, Joris Hoeks

**Affiliations:** 1 Department of Nutrition and Movement Sciences, NUTRIM Institute of Nutrition and Translational Research in Metabolism, Maastricht University, Maastricht, the Netherlands; 2 Department of Radiology and Nuclear Medicine, NUTRIM Institute of Nutrition and Translational Research in Metabolism, Maastricht University Medical Centre, Maastricht, the Netherlands; 3 Division of Neurology, Department of Medicine, Faculty of Medicine and Health Sciences, Université de Sherbrooke, Sherbrooke, QC Canada; 4 Centre de Recherche du Centre Hospitalier Universitaire de Sherbrooke, Sherbrooke, QC Canada; 5 Institute for Clinical Diabetology, German Diabetes Center, Leibniz Institute for Diabetes Research at Heinrich Heine University Düsseldorf, Düsseldorf, Germany; 6 German Center for Diabetes Research (DZD e.V.), Partner Düsseldorf, Neuherberg, Germany; 7 Department of Clinical Epidemiology, Leiden University Medical Centre, Leiden, the Netherlands

**Keywords:** Cold, Continuous glucose monitoring, Glucose homeostasis, Lifestyle intervention, Metabolism, Prediabetes, Type 2 diabetes

## Abstract

**Aims/hypothesis:**

Repeated cold exposure with shivering has been proposed as a potential strategy to enhance glucose metabolism by increasing energy expenditure and substrate utilisation. However, acute effects/benefits of cold-induced shivering on glucose homeostasis in metabolically compromised individuals are unknown. Here, we aimed to determine whether cold exposure at two different intensities improves 24 h glucose homeostasis in individuals with prediabetes and type 2 diabetes.

**Methods:**

In a randomised crossover trial conducted in the South Limburg/Maastricht region of the Netherlands, men and postmenopausal women with prediabetes (*n*=12) and stable type 2 diabetes (*n*=12), aged 40–75 years, body mass index ≥27 and ≤35 kg/m^2^, non-smoking and sedentary, underwent two whole-body cold exposure sessions using a water-perfused suit. Session order was randomised using an online randomisation tool (randomizer.org); participants were masked to the cold exposure intensity received, but investigators were not. Sessions were designed to elicit ~1.5-fold (mild, 15°C) and ~2.5-fold (moderate, 4°C) increases in resting metabolic rate (RMR). Continuous glucose monitoring assessed interstitial glucose concentrations over 24 h periods before and after each intervention, with controlled diet and activity. Shivering was confirmed via indirect calorimetry and electromyography.

**Results:**

In both study groups and periods, RMR increased significantly vs baseline (*p*<0.001 for all). In prediabetes, the increase in the final 1 h of cold was 1.53 × RMR in mild and 1.94 × RMR in moderate cold. In type 2 diabetes, the increase was 1.57 × RMR and 2.09 × RMR in the final 1 h of mild and moderate cold, respectively. In prediabetes, neither mild nor moderate cold exposure altered mean 24 h glucose levels. In contrast, after mild cold exposure the type 2 diabetes group exhibited a significant reduction in mean 24 h glucose levels (−0.6 ± 0.5 mmol/l, *p*=0.003) and fasting glucose (−0.6 ± 0.8 mmol/l, *p*=0.019), as well as an increase in time in normal range (+8.8 ± 10.3%, *p*=0.013) and reduced time in hyperglycaemia (−10.9 ± 12.9%, *p*=0.014). Moderate cold did not significantly affect any of the glucose outcomes in type 2 diabetes. Baseline fasting glucose, age and ALT levels were predictors of the glucose-lowering response, suggesting greater benefits in individuals who have higher baseline glucose levels, are younger and/or have more optimal liver health, i.e. lower ALT.

**Conclusions/interpretation:**

Acute mild cold exposure with shivering reduced 24 h glucose levels in individuals with type 2 diabetes. No changes were observed in prediabetes. The observed effects appear to depend on baseline metabolic status rather than acute substrate utilisation during cold exposure. These findings support the potential of cold exposure as an adjunct non-pharmacological therapy for type 2 diabetes, although further mechanistic studies and validation in larger cohorts are warranted.

**Trial registration:**

ClinicalTrials.gov NCT05576025

**Funding:**

Dutch Organisation for Knowledge and Innovation in Health, Healthcare and Well-being (ZonMw): 09120012010062

**Graphical Abstract:**

**Figure:**
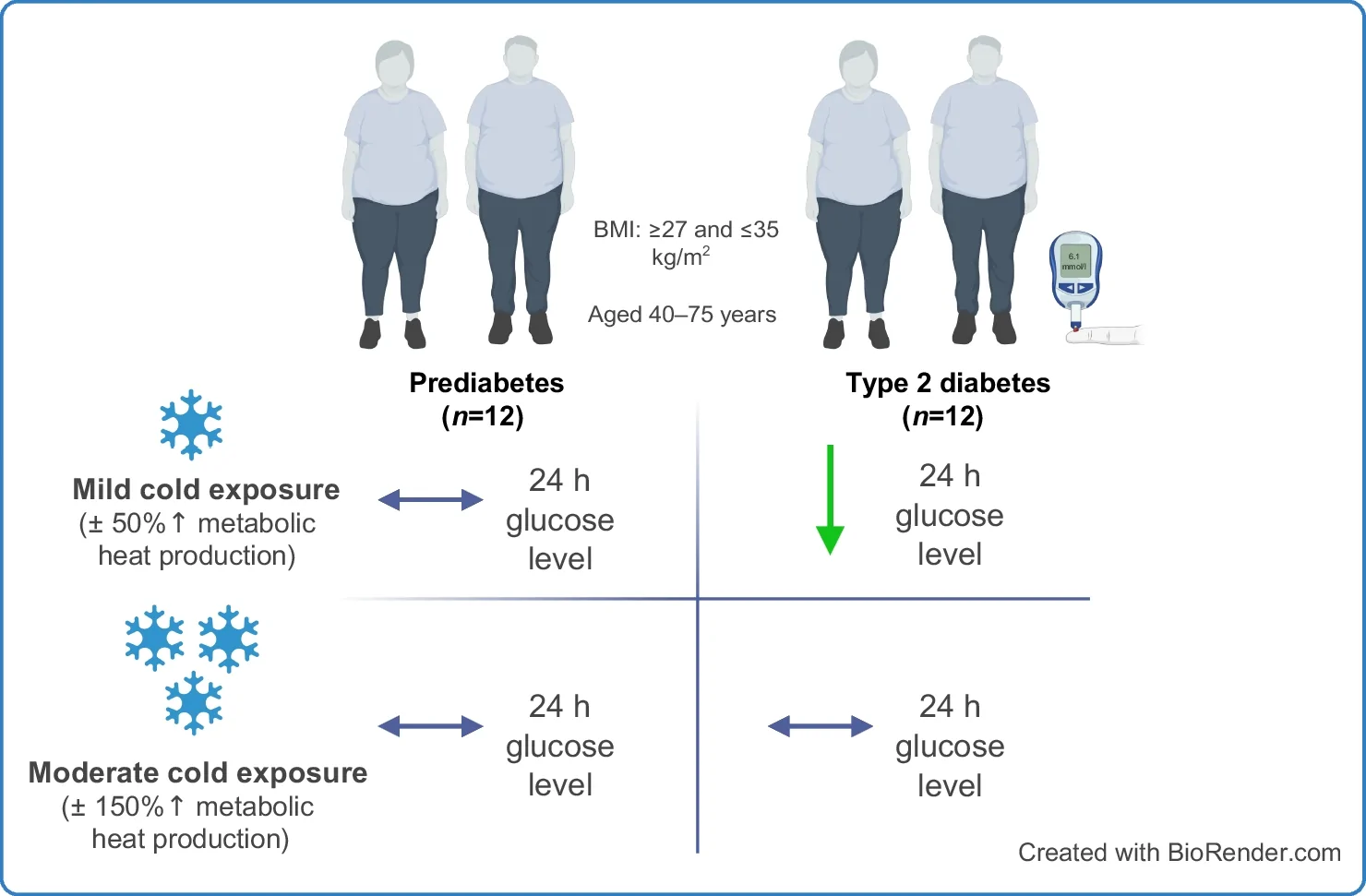

**Supplementary Information:**

The online version contains peer-reviewed but unedited supplementary material available at https://doi.org/10.1007/s00125-026-06809-z.

**Figure:**
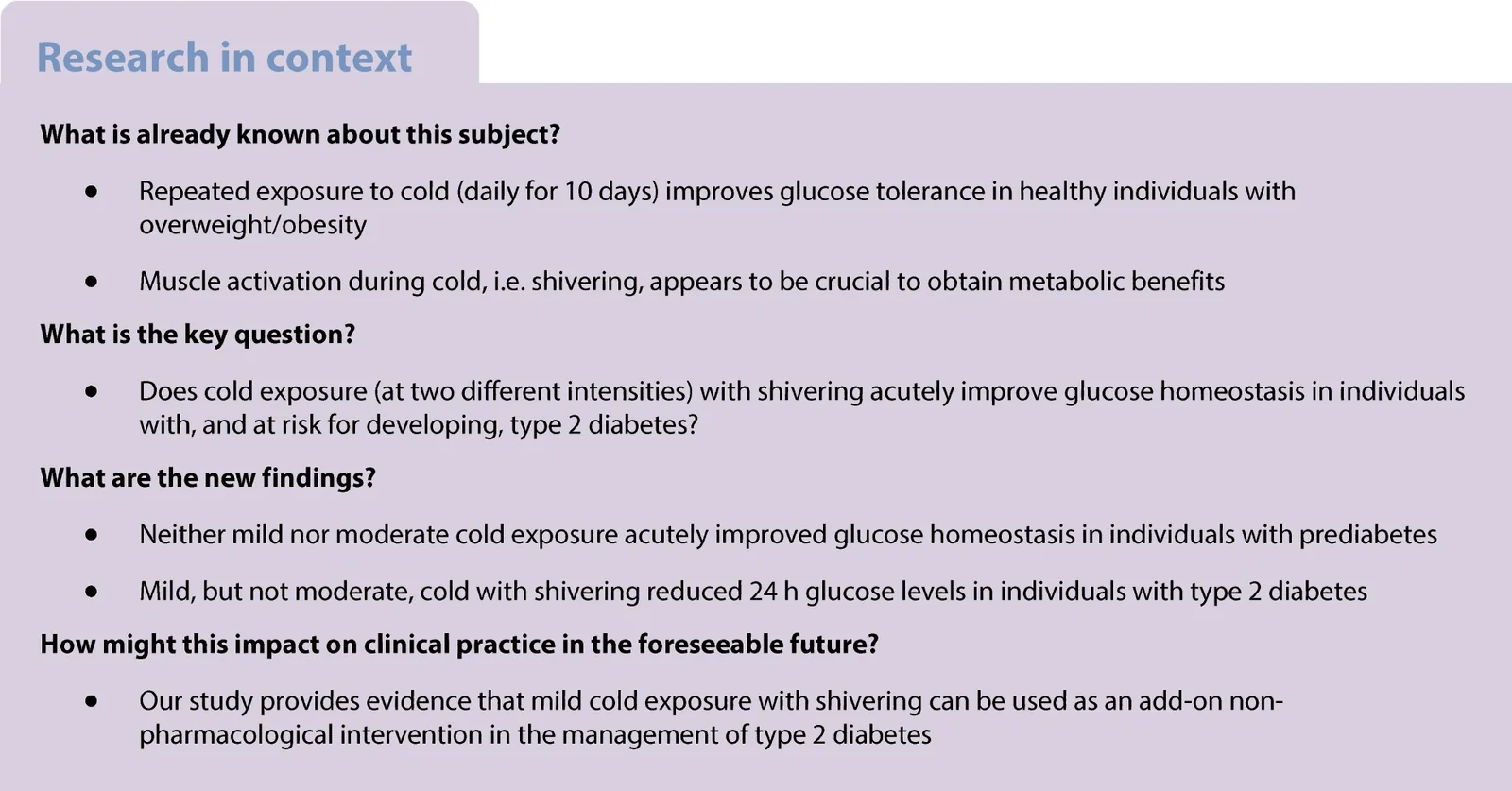

## Introduction

In 2021, an estimated 537 million adults suffered from diabetes, of which 90% was classified as type 2 diabetes [1]. Sedentary lifestyle, excessive caloric intake and ageing are among the top contributors to its rising prevalence [2]. Lifestyle modifications including exercise and a balanced diet are highly effective strategies for type 2 diabetes prevention and management, yet long-term adherence is often suboptimal [3], highlighting a need to explore complementary approaches to promote metabolic health.

Evidence increasingly suggests that cold exposure, particularly at temperatures inducing muscle contractions through shivering, may improve metabolic health. We recently showed that a 10 day cold acclimation protocol eliciting a ~1.7-fold increase in resting energy expenditure (EE) during cold exposure improved glucose tolerance, fasting glucose and lipid profiles of healthy individuals with overweight/obesity [4], with fasting plasma glucose already reduced after a single cold exposure session [4]. Whether acute cold exposure improves 24 h glucose homeostasis in people with metabolic dysregulations, such as impaired glucose tolerance and type 2 diabetes, remains to be established.

Mechanistically, cold exposure mobilises glucose and lipid utilisation to sustain metabolic thermogenesis via sympathetic stimulation [5, 6]. At cold exposure intensities eliciting shivering thermogenesis, skeletal muscle substrate utilisation increases substantially in both healthy [6, 7] and metabolically compromised individuals [8]. While fat oxidation dominates at milder intensities of shivering thermogenesis [9], the relative contribution of carbohydrate (CHO) oxidation (specifically, fuelled by intramuscular glycogen) becomes predominant upon more severe cold stress closer to and above 2.5 × resting metabolic rate (RMR), at least in lean, healthy individuals [9, 10]. Whether different cold intensities differentially impact substrate utilisation and 24 h glucose homeostasis in metabolically compromised individuals remains unclear.

To address these gaps, we investigated the acute effects of mild and moderate cold exposure on 24 h glucose levels in people with type 2 diabetes and prediabetes. The primary aim was to assess the impact of acute cold exposure on mean 24 h glucose in both groups; the secondary aim was to determine whether the magnitude of effect differed between the two cold intensities. We hypothesised that mild and moderate cold exposure would reduce mean 24 h glucose in both groups, with a larger reduction in moderate cold.

## Methods

### Participants

**Fig. 1:**
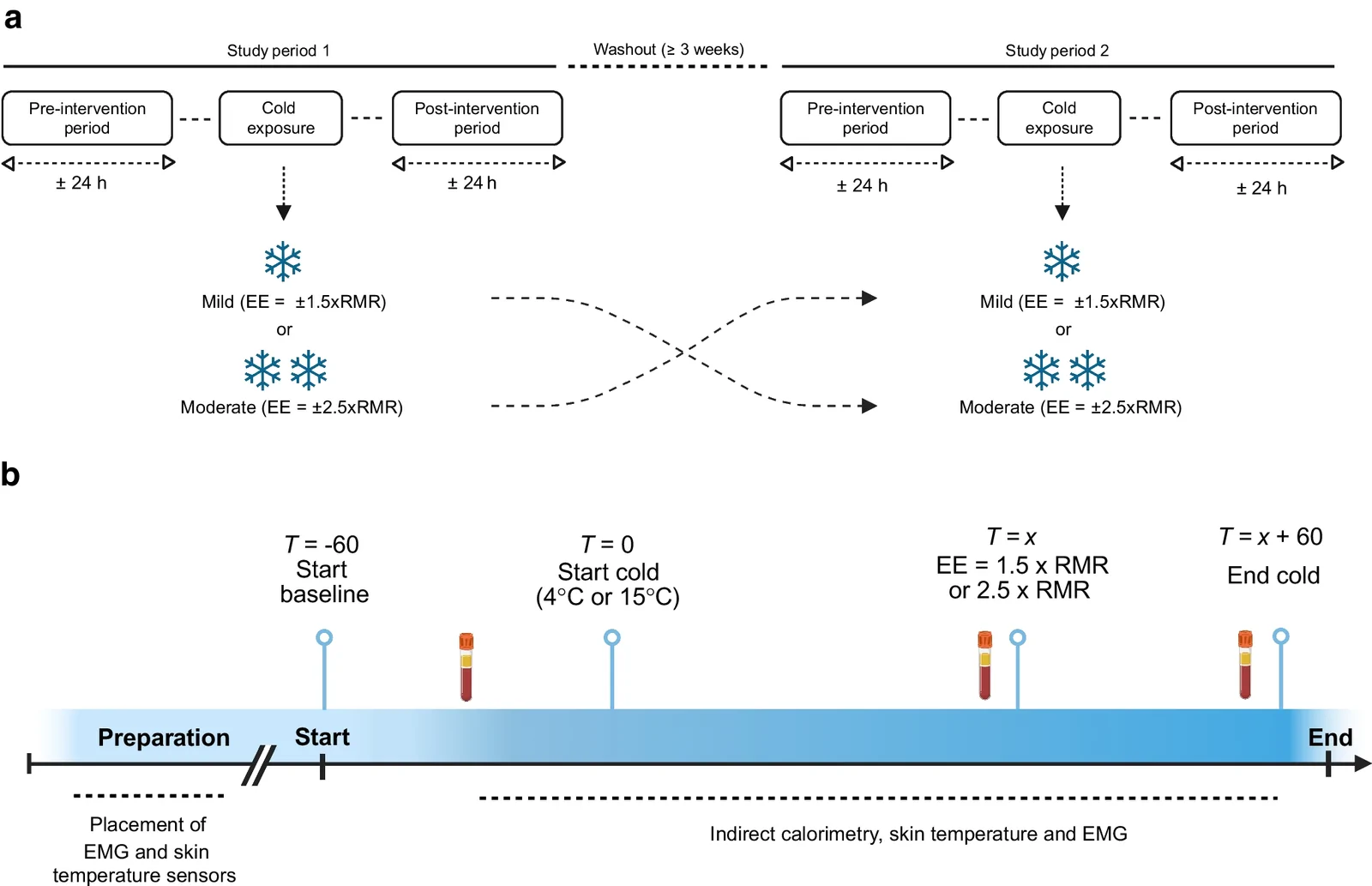
Study design (**a**) and overview of the individualised cold exposure protocol (**b**). In a crossover design, participants were exposed to cold at mild and moderate intensities with  ±1.5-fold and  ±2.5-fold increase in RMR, respectively. Interstitial glucose concentrations were measured for  ±24 h before and after each cold exposure protocol (**a**). Each cold exposure was preceded by a 1 h baseline period before cold exposure at either 15°C (mild) or 4°C (moderate) began. Upon a 1.5-fold or 2.5-fold increase in RMR, the final 1 h of cold exposure began. Plasma samples were obtained 30 min into the baseline period, and at the onset and end of the final 1 h of cold exposure. EE, substrate oxidation, skin temperature and muscle activity were measured at baseline and during cold exposure

The study included 12 participants with prediabetes and 12 with type 2 diabetes, all sedentary and unacclimated to the cold (electronic supplementary material [ESM] Fig. 1). Race and ethnicity data were not collected during recruitment; the study was conducted at a single centre in Maastricht, the Netherlands, and participants were drawn from the population in the Limburg region, which is predominantly of White Northern European background, limiting generalisability of our results to other ethnic groups. Participants were non-smoking men or postmenopausal women (≥1 year with no hormone replacement therapy), aged 40–75 years, with body mass index (BMI) 27–35 kg/m^2^, stable weight for ≥3 months (≤5 kg change) and no active cardiovascular disease, anaemia or alcohol/substance use disorder.

Prediabetes was determined via an oral glucose tolerance test (OGTT) performed following a ≥10 h overnight fast. Participants were classified as having prediabetes if one or a combination of the following criteria applied to them: (1) impaired glucose tolerance, defined as plasma glucose ≥7.8 and ≤11.1 mmol/l 2 h after glucose drink consumption during OGTT; (2) fasting plasma glucose ≥6.1 and ≤6.9 mmol/l; (3) glucose clearance rate (oral glucose insulin sensitivity [OGIS]) ≤360 ml/kg/min; and/or (4) HbA_1c_ levels between 39 mmol/mol (5.7%) and 46 mmol/mol (6.4%). They were excluded in the case of prior diagnosis of type 2 diabetes.

Type 2 diabetes participants were diagnosed ≥1.5 years prior and had well-controlled glucose (HbA_1c_ <69 mmol/mol [8.5%]), no active comorbidities and stable medication for ≥3 months. Insulin and SGLT2 inhibitor users were excluded; other medications were allowed if non-interfering. As an adjunct therapy, participants continued all prescribed treatments throughout the study. The study was conducted at the Metabolic Research Unit, Maastricht University, between 30 January 2023 and 28 November 2024.

### Ethical approval

The study was conducted in accordance with the declaration of Helsinki and was approved by the Medical Ethical Committee of Maastricht University Medical Centre. The study was registered at ClinicalTrials.gov (registration no. NCT05576025). All participants provided their written informed consent prior to the start of the screening.

### Study design

In a randomised crossover design, all participants were exposed to two cold exposure sessions with different intensities, in a single-blind fashion, with a wash-out period of ≥3 weeks (Fig. 1a). Randomisation was performed using an online randomiser (randomizer.org). To investigate the effects on glucose homeostasis, interstitial glucose concentrations were continuously measured on the days before, during and after the cold exposure. Body composition was determined on the first day of the first study period, in the fasted state, using air displacement plethysmography (BodPod, Cosmed, Italy).

### Standardisation procedures

Standardised meals were provided during the study outcome window. Total daily energy needs were calculated by multiplying basal metabolic rate (BMR), estimated with the Harris–Benedict formula [11], by a physical activity level (PAL): 1.8 (<50 years), 1.7 (50–75 years) and 1.6 (70–75 years) [12]. Participants could consume  ±10% of estimated needs to ensure satiety. Meals reflected a typical Dutch diet: ~20–25% of daily calories at breakfast and lunch, ~40–45% at dinner and up to 10% from snacks, with 45–50% CHO, 30–35% fat and 10–15% protein. Participants avoided alcohol and caffeine before, during and after cold exposure. Compliance was monitored using food diaries. Participants were instructed to avoid exercise and intense physical activity. Triaxial physical activity loggers (ActivPal3, PAL Technologies) were used to ensure adherence [13].

### Cold exposure

Whole-body cold exposure was induced using a liquid-conditioned suit (LCS; Three Piece, Med-Eng, Canada) with water temperature controlled by cooling towers (Blanketrol III, Gentherm, USA). During baseline, participants rested supine at a room temperature of ~21.0°C without water circulation. Subsequently, room temperature was reduced to ~17.0°C and LCS water temperature was set to 15°C (mild) or 4°C (moderate). Shivering was verified by indirect calorimetry, surface electromyography (EMG) and visual inspection. Exposure duration was individualised based on metabolic heat production; cooling continued until RMR increased ~1.5-fold (mild) or ~2.5-fold (moderate), followed by an additional 1 h. Blood was collected 30 min into baseline and at the start and end of the final hour of cold exposure (Fig. 1b).

Skin and core temperatures were measured throughout the baseline period and cold exposure as previously described [4]. Skeletal muscle activity during cold exposure was measured by means of surface EMG electrodes (Trigno, Delsys, USA) placed on top of six muscle groups on the right side of the body, namely pectoralis major, trapezius upper, latissimus dorsi, vastus lateralis, vastus medialis and rectus femoris, as previously described [4].

### EE and substrate oxidation

Whole-body O_2_ consumption and CO_2_ production were measured at 1 min intervals using an automated respiratory analyser and ventilated hood system (Omnical, Maastricht University, the Netherlands) to determine EE and substrate oxidation during the final 30 min of the baseline period and throughout each cold exposure. EE was calculated according to Weir’s formula, whereby protein oxidation was estimated to be 12.4% of resting EE [14]. Thereafter, CHO and fat oxidation were calculated according to Brouwer’s equation [15].

### Continuous glucose monitoring

Interstitial glucose concentrations were measured over  ±24 h periods at 15 min intervals before and after each cold exposure via a continuous glucose monitor (CGM) (FreeStyle Libre Pro iQ, Abbott Laboratories, USA) placed on the right upper arm (triceps brachii area) of each participant. Due to technical failure, CGM data of one participant with prediabetes (for both study periods) and one participant with type 2 diabetes (for moderate cold period) were lacking, and hence not included in the analyses.

The pre- and post-intervention periods were defined as ~24 h windows spanning all daily meals, from lunch on the first day of each study period, and from lunch after each cold exposure session, respectively, and ending 2 h after the breakfast on the following day (see ESM Fig. 2 for more details). Mean durations of pre- and post-intervention time windows are presented in ESM Fig. 3. To avoid the confounding effects of a missed breakfast on the cold exposure day, participants were asked to skip breakfast on the pre-intervention day as well.

Mean diurnal (06:00 to 00:00 hours), nocturnal (00:00 to 06:00 hours) and fasting glucose (06:00 to 07:00 hours), as well as the percentage of time participants spent within three glucose ranges, defined as time in: (1) normal glucose range (4.4–7.2 mmol/l); (2) high glucose range (7.3–9.9 mmol/l); and (3) hyperglycaemia (>10.0 mmol/l) [16], were calculated within the same time windows used to calculate pre- and post-intervention means.

### Plasma substrate analyses

Blood samples were collected and processed as previously described [4]. Plasma glucose (Horiba, Montpellier, France) and serum non-esterified fatty acid (NEFA) (Instruchemie, Groningen, the Netherlands) concentrations were determined via a colorimetric assay performed in duplicate using a Pentra C400 spectrophotometer (Horiba, Montpellier, France). Serum triglyceride (TG) (Roche Diagnostics, Basel, Switzerland) concentrations were determined by spectrophotometric analysis on the COBAS PRO spectrophotometer. Plasma insulin concentration was determined in duplicate by enzyme immunoassay (Crystal Chem, Elk Grove Village, USA). Plasma glycerol was measured by gas chromatography–mass spectrometry using selected ion recording (SIR), as previously described [17].

### Sample size

The primary aim was to assess whether acute cold exposure with shivering reduces 24 h glucose AUC in individuals with prediabetes and type 2 diabetes. Sample size calculations were performed separately for each group, and the larger required sample size was applied to both to ensure adequate power.

For prediabetes, a 15% reduction in 24 h glucose AUC was expected. Based on CGM data from a comparable crossover study [18], within-subject SD was 1123.4 mmol/l × min. With 80% power and α=0.05 (two-sided paired *t* test), ten participants were required. For type 2 diabetes, a larger 20% reduction was anticipated. Using CGM data from a similar crossover study [19], within-subject SD was 2170.2 mmol/l × min. Thus, 12 participants were required. To ensure adequate power for both groups, 12 participants were included in each group.

### Statistical analyses

Data are presented as mean ± SD unless stated otherwise. Analyses and visualisation were performed using GraphPad Prism (v8.4.3). Normality was assessed with the Shapiro–Wilk test. Paired *t* tests or Wilcoxon signed rank tests assessed pre–post differences and comparisons between baseline vs final hour and mild vs moderate cold. Changes in plasma substrates were analysed using repeated-measures ANOVA or non-parametric equivalents with assumed sphericity and Bonferroni-corrected post hoc tests. Multiple linear regression evaluated associations between changes in 24 h glucose and participant characteristics, with model fit assessed by adjusted *R*^2^, AIC and VIF, and pairwise correlation was tested using Pearson (parametric) or Spearman (non-parametric) analyses, corrected for multiple testing via the Holm–Sidak test. Statistical significance was set at *p*<0.05.

## Results

### Participant characteristics

Individuals with prediabetes (*n*=12, men/women: 6/6) and type 2 diabetes (*n*=12, men/women: 9/3) participated in this study (Table 1). Age and BMI were comparable between the groups (*p*=0.469 and *p*=0.529, respectively). Individuals with prediabetes were characterised by a higher fat mass (FM) (*p*=0.021) and a lower fat-free mass (FFM) (*p*=0.021) compared with type 2 diabetes. When adjusted for age and sex based on reference values [20], difference in FM was not significant (*p*=0.139) but the difference in FFM remained (*p*=0.019). As expected, mean plasma HbA_1c_ and fasting glucose levels were higher in type 2 diabetes vs prediabetes (*p*<0.001), with no difference in fasting insulin levels (*p*=0.787). Ten participants with type 2 diabetes were on oral glucose-lowering medication treatment and two participants were on dietary control only (ESM Table 1).

**Table 1:** Participant characteristics

Variable (unit)	Prediabetes group (*n*=12)	T2D group (*n*=12)	*p* value
Age (years)	62.2 ± 10.1	64.8 ± 7.4	0.469
Male/female	6/6	3/9	NA
BMI (kg/m^2^)	31.8 ± 4.4	30.8 ± 3.1	0.529
Weight (kg)	94.88 ± 17.37	93.46 ± 14.98	0.832
Height (m)	1.72 ± 0.07	1.74 ± 0.11	0.696
BSA (m^2^)	2.07 ± 0.20	2.07 ± 0.22	0.977
FM (%)	41.83 ± 7.11	34.07 ± 7.78	0.021*
FFM (%)	58.17 ± 7.16	65.93 ± 7.78	0.021*
SBP (mmHg)	140.40 ± 18.65	145.90 ± 13.75	0.420
DBP (mmHg)	89.14 ± 8.33	90.92 ± 8.44	0.608
Fasting glucose (mmol/l)	5.3 ± 0.4	8.0 ± 1.9	<0.001***
Fasting insulin (pmol/l)	83.4 ± 66.1	79.7 ± 43.6	0.787
HbA_1c_ (mmol/mol)	37.2 ± 3.8	51.0 ± 9.4	<0.001***
HbA_1c_ (%)	5.6 ± 0.4	6.8 ± 0.8	<0.001***
2 h glucose during OGTT (mmol/l)	8.26 ± 2.07	NA	NA
OGIS (ml min^−1^ m^−2^)	333.10 ± 41.92	NA	NA

Data are presented as mean ± SD. Differences between the two study groups were assessed with an unpaired *t* test or the non-parametric equivalent

Statistical differences are denoted as *p<0.05, **p<0.01, ***p<0.001

BSA, body surface area; DBP, diastolic blood pressure; SBP, systolic blood pressure; T2D, type 2 diabetes

### Caloric intake and PALs are comparable during the pre- vs post-intervention time windows

Before vs after mild cold, daily caloric intake was similar in both prediabetes (*p*=0.117) and type 2 diabetes (*p*=0.974). Similarly, pre and post moderate cold, daily caloric intake was not different in prediabetes (*p*=0.282) and type 2 diabetes (*p*=0.436). Macronutrient composition of meals was also comparable across study arms and pre vs post cold exposure within each period (*p*>0.05) (ESM Table 2). Before vs after mild and moderate cold exposure, study groups spent comparable amounts of time in the three main physical activity categories, namely, sitting, standing and stepping (*p*>0.05, ESM Table 3).

### Physiological and metabolic responses to the mild and moderate cold exposure

Total duration of the cold exposure sessions was comparable between groups (*p*>0.05), but both groups spent significantly more time in moderate vs mild cold (*p*<0.05).

**Fig. 2:**
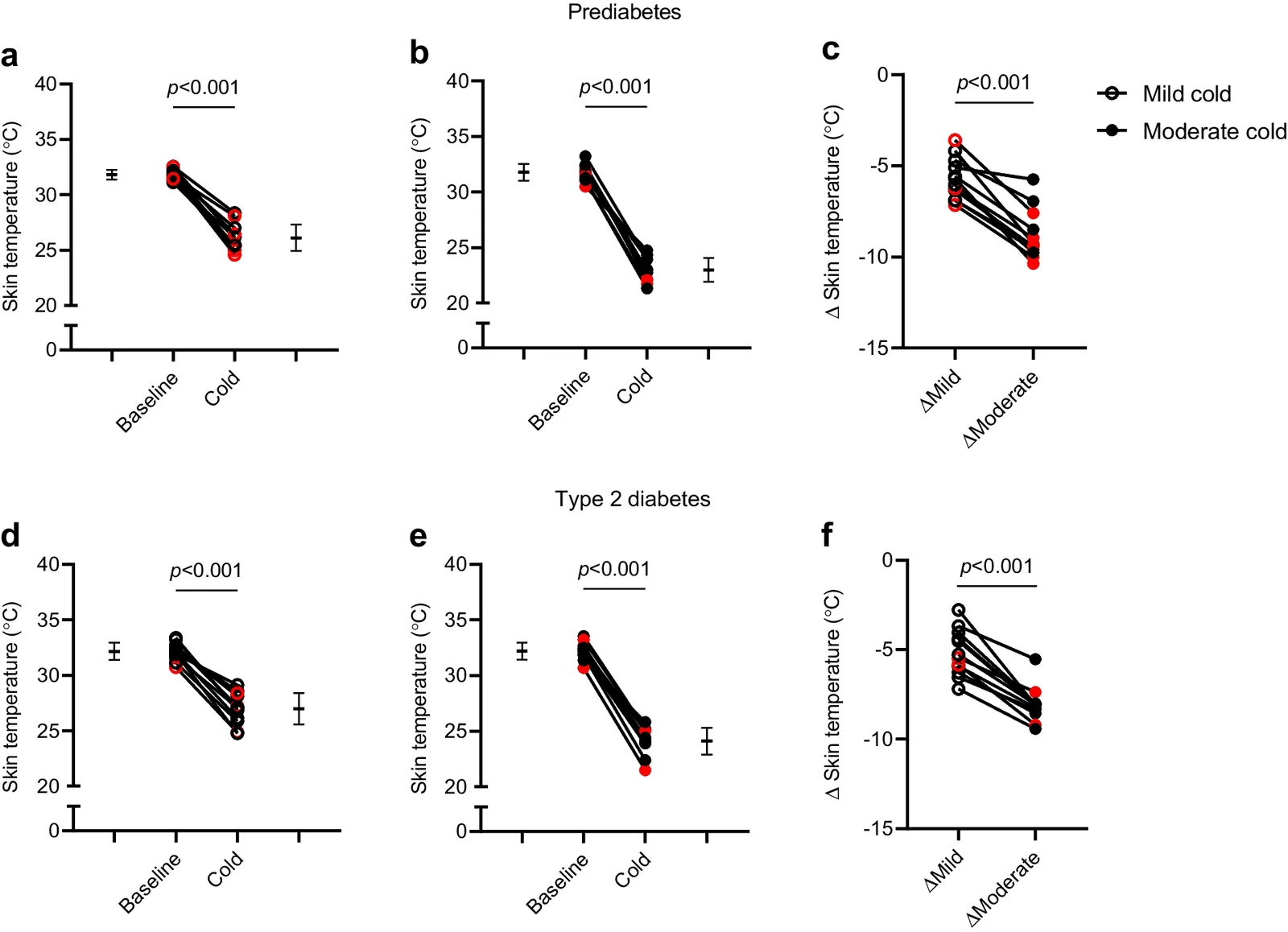
Mean skin temperature during baseline and the final 1 h of mild and moderate cold exposure, and the magnitude in reduction (i.e. Δ post–pre) induced by mild vs moderate cold. Group means are presented as mean ± SD and individual data points are plotted for the prediabetes (**a**–**c**) and type 2 diabetes (**d**–**f**) groups (black dots for men and red dots for women). All data were analysed with paired *t* test or Wilcoxon signed rank test (*n*=12). Statistical significance was deemed as *p*<0.05

**Fig. 3:**
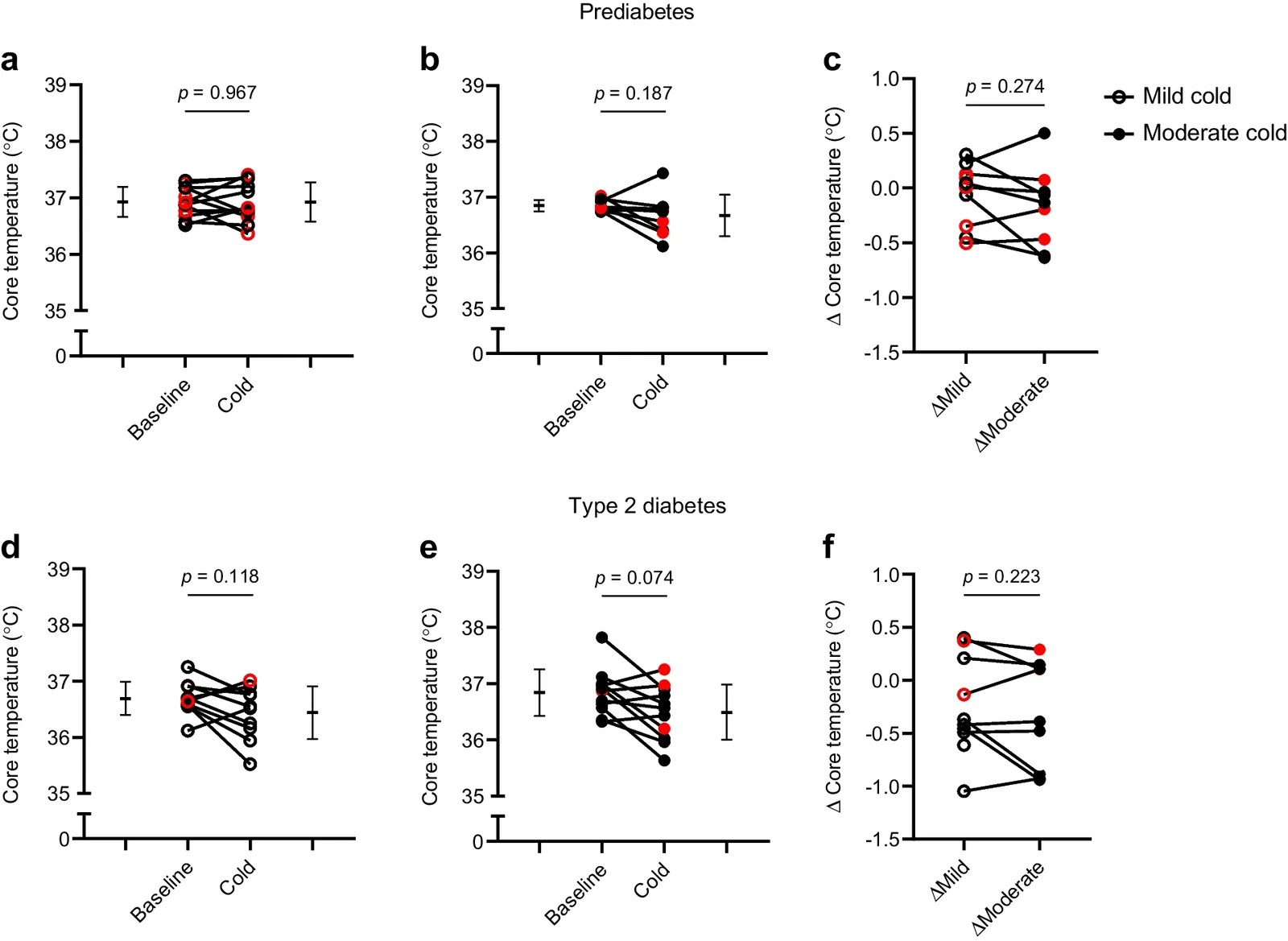
Mean core temperature during baseline and the final 1 h of mild and moderate cold exposure, and the magnitude in changes induced by mild vs moderate cold (i.e. Δ post–pre). Group means are presented as mean ± SD and individual data points are plotted for the prediabetes (**a**–**c**) and type 2 diabetes (T2D) groups (**d**–**f**) (black dots for men and red dots for women). All data were analysed with paired *t* test or Wilcoxon signed rank test. *n*=12 for prediabetes, mild; *n*=9 for prediabetes, moderate; *n*=8 for T2D, mild; *n*=11 for T2D, moderate. Statistical significance was deemed as *p*<0.05

**Fig. 4:**
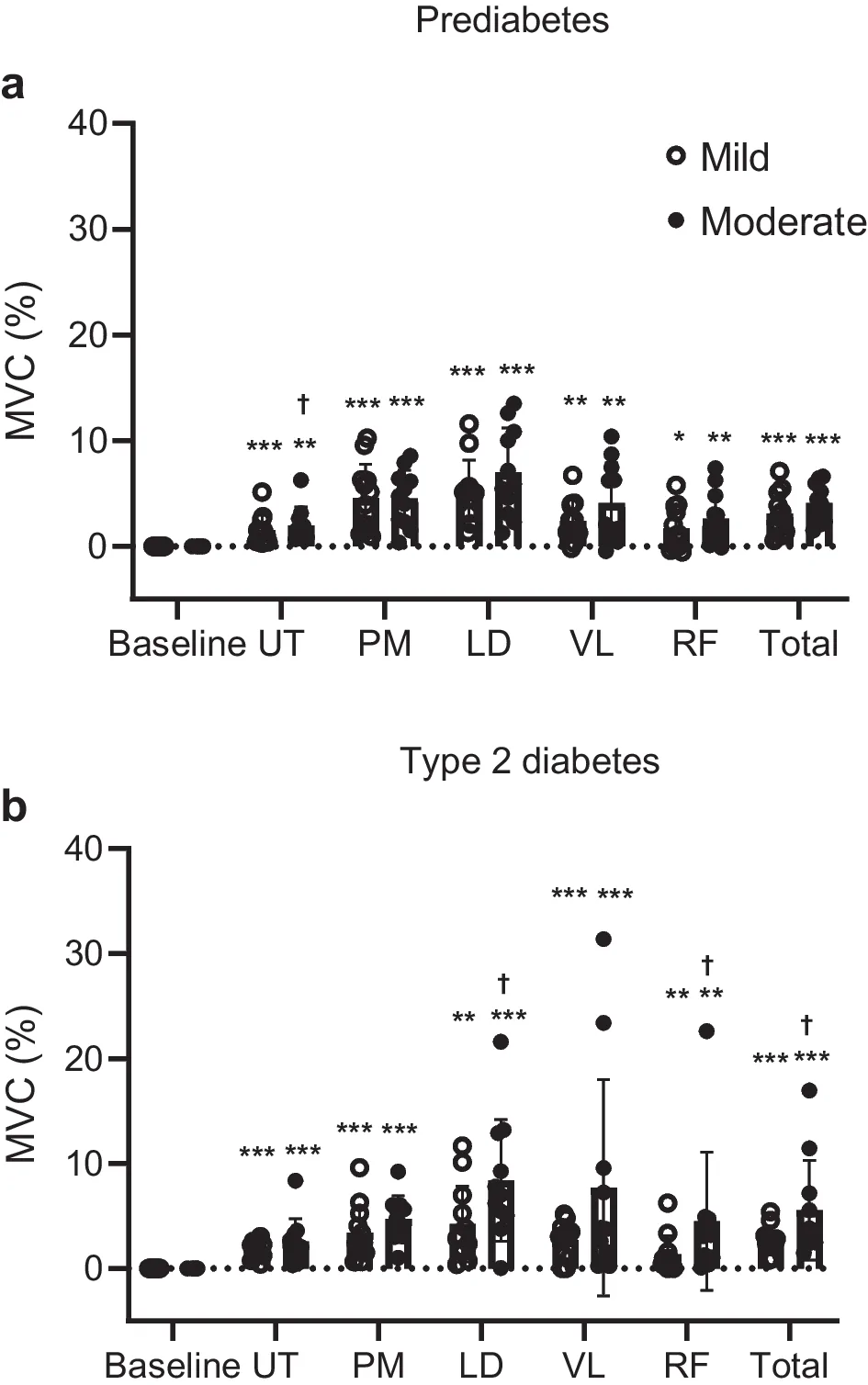
Skeletal muscle activity in the prediabetes (**a**) and type 2 diabetes (**b**) groups at baseline and in the final 1 h of mild (white dots) and moderate (black dots) cold exposure in five muscle groups, namely the upper trapezius (UT), pectoralis major (PM), latissimus dorsi (LD), vastus lateralis (VL) and rectus femoris (RF), and mean of all muscle groups combined (total). Values are expressed relative to MVC (%MVC). Statistical differences were tested with paired *t* test or non-parametric equivalent compared with the respective baseline period (*p<0.05, **p<0.01, ***p<0.001) or with the alternative cold exposure intensity (^†^*p*<0.05 vs mild). Data are presented as mean ± SD

The mean skin temperature decreased in the final 1 h of cold exposure and this reduction was more pronounced during moderate vs mild cold in both groups (*p*<0.001) (Fig. 2). Core temperature remained unaltered during both cold exposure sessions (Fig. 3) and remained above the hypothermic threshold value of 35.0°C. In both prediabetes and type 2 diabetes*,* upon both mild and moderate cold exposure, skeletal muscle activity in the final 1 h of cold exposure was significantly higher when compared with the corresponding baseline (Fig. 4). In type 2 diabetes, latissimus dorsi and rectus femoris demonstrated higher activity during moderate vs mild cold (*p*<0.05 for both muscles). In prediabetes and in the remaining muscles in type 2 diabetes, activity was comparable between cold intensities.

In prediabetes, mean EE rate during the final 1 h of cold exposure was 1.53-fold higher (i.e. 1.53 × RMR) (*p*<0.001) upon mild and 1.94-fold higher (i.e. 1.94 × RMR) upon moderate cold (*p*<0.001) vs the respective baseline. In type 2 diabetes, EE increased by 1.57-fold (i.e. 1.57 × RMR) (*p*<0.001) and 2.09-fold (i.e. 2.09 × RMR) (*p*<0.001) during the final 1 h of mild and moderate cold, respectively. In both groups, the increase in EE was significantly higher during moderate vs mild cold (*p*<0.01) (Table 2).

**Table 2:** EE and substrate oxidation at baseline and in the final 1 h of cold exposure

Variable	EE (kJ/min)		CHO oxidation (g/min)		Fat oxidation (g/min)		CHO oxidation (%)		Fat oxidation (%)		RER	
	Baseline	Cold	Baseline	Cold	Baseline	Cold	Baseline	Cold	Baseline	Cold	Baseline	Cold
Prediabetes group												
Mild	4.88 ± 0.80	7.48 ± 1.28***	0.08 ± 0.05	0.12 ± 0.07*	0.07 ± 0.03	0.11 ± 0.03***	27.7 ± 17.6	25.7 ± 14.1	54.2 ± 17.5	56.2 ± 14.0	0.805 ± 0.052	0.799 ± 0.041
Moderate	4.70 ± 0.88	9.10 ± 2.35***/^††^	0.10 ± 0.05	0.16 ± 0.10**/^†^	0.06 ± 0.02	0.13 ± 0.03***	33.9 ± 16.6	27.7 ± 11.2	48.1 ± 16.5	54.2 ± 11.1	0.823 ± 0.049	0.805 ± 0.033
T2D group												
Mild	5.13 ± 0.65	8.07 ± 1.56***	0.11 ± 0.02	0.13 ± 0.05	0.06 ± 0.02	0.12 ± 0.03***	38.1 ± 9.6	27.1 ± 7.9**	43.9 ± 9.5	54.8 ± 7.8**	0.836 ± 0.029	0.803 ± 0.023**
Moderate	5.10 ± 0.84	10.59 ± 2.63***/^††^	0.10 ± 0.05	0.19 ± 0.09*/^†^	0.07 ± 0.03	0.15 ± 0.05***/^†^	33.7 ± 16.5	29.2 ± 10.1	48.3 ± 16.4	52.8 ± 10.1	0.827 ± 0.049	0.809 ± 0.030

Data are presented as mean ± SD

Paired *t* test or non-parametric equivalent was used to test for differences

Statistical differences are denoted as *p<0.05, **p<0.01, ***p<0.001 vs respective baseline; ^†^*p*<0.05, ^††^*p*<0.01 vs mild cold

RER, respiratory exchange ratio; T2D, type 2 diabetes

In prediabetes, absolute rates of both CHO and fat oxidation increased during both mild and moderate cold exposure, with no significant change in relative contributions (Table 2). In type 2 diabetes, only the absolute rate of fat oxidation increased in the final 1 h of mild cold, whereas the absolute rates of both CHO and fat oxidation increased upon moderate cold exposure vs baseline (Table 2). In both groups, the relative contribution of each substrate was not different between the final 1 h of mild vs moderate cold exposure, with predominantly fat oxidation contributing to EE (Table 2).

**Fig. 5:**
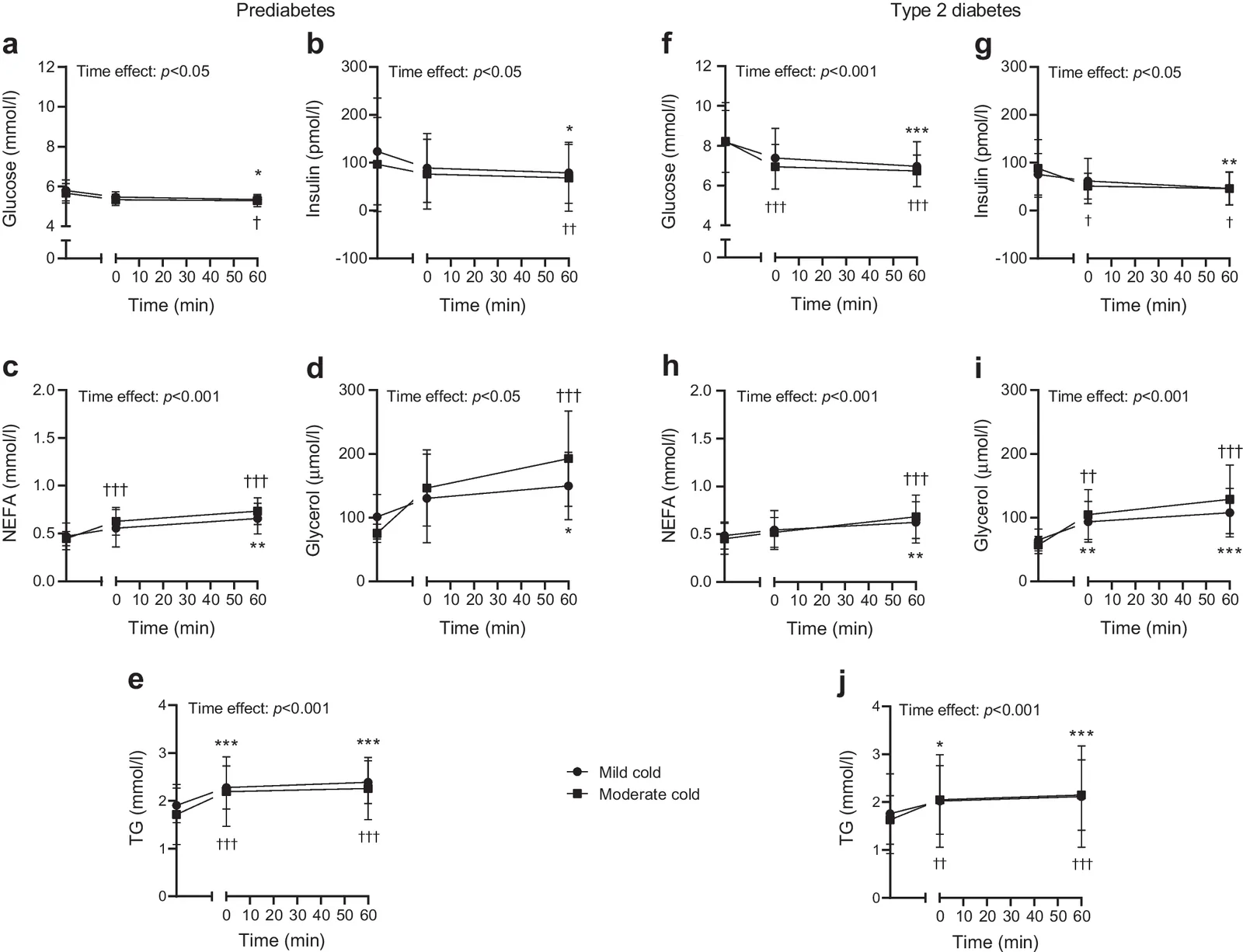
Plasma substrate concentrations at baseline and during mild and moderate cold exposure in the prediabetes (**a**–**e**) and type 2 diabetes (T2D) (**f**–**j**) groups. Data were analysed with repeated-measures ANOVA or non-parametric equivalent. Data at the three time points, i.e. baseline, onset and end of the final 1 h of cold exposure, are expressed as mean ± SD. *n*=9 for prediabetes, mild and T2D, moderate (*n*=8 for TG only for T2D, moderate)*; n*=10 for prediabetes, moderate and T2D, mild*.* Statistical differences are denoted as *p<0.05, **p<0.01 and ***p<0.001 vs baseline of mild cold; ^†^*p*<0.05, ^††^*p*<0.01 and ^†††^*p*<0.001 vs baseline of moderate cold

In both groups, plasma glucose and insulin concentrations decreased significantly during both mild and moderate cold exposure as compared with baseline (time effect: *p*<0.05). On the other hand, plasma glycerol, NEFA and TG levels increased during both cold exposure sessions, in both prediabetes and type 2 diabetes (Fig. 5, ESM Table 4). To estimate whether lipolysis is matched by coordinated fatty acid release and downstream lipid handling, we next assessed how cold-induced changes in glycerol associated with changes in plasma NEFA and TG. We pooled the groups’ data and performed a linear regression analysis with diabetes status as a covariate, and observed that the fold increase in glycerol at the end of the mild cold exposure relative to baseline was correlated with the fold increase in NEFA (*r*^2^=0.63, *p*=0.007) and TG (*r*^2^=0.50, *p*=0.042) levels. Such correlations were not observed upon moderate cold (*r*^2^=0.22, *p*=0.352 and *r*^2^=0.18, *p*=0.470 for NEFA and TG, respectively).

### Mild cold exposure reduces 24 h glucose levels in individuals with type 2 diabetes, but glucose levels remain unaffected in prediabetes

**Fig. 6:**
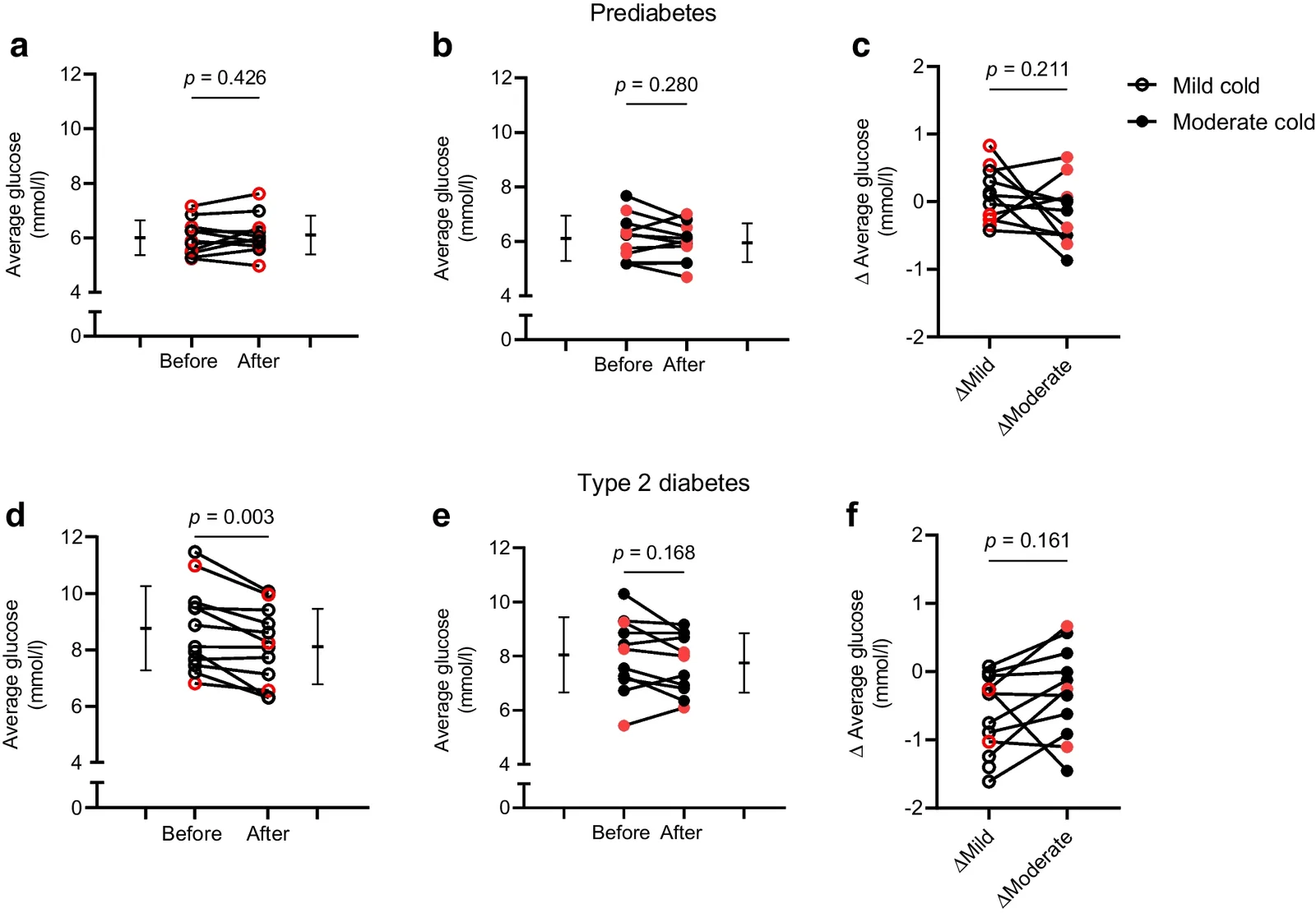
Mean meal-to-meal interstitial glucose levels, obtained before and after mild and moderate cold exposure from participants with prediabetes (**a**, **b**, *n*=11) and with type 2 diabetes (**d**, **e**, mild: *n*=12; moderate: *n*=11). (**c**, **f**) Δ Mean represents the difference in mean glucose concentrations before vs after for the respective cold intensity. All data were analysed with paired *t* test or Wilcoxon signed rank test. Group means are presented as mean ± SD and individual data points are plotted for men (black dots) and women (red dots). Statistical significance was deemed as *p*<0.05

**Fig. 7:**
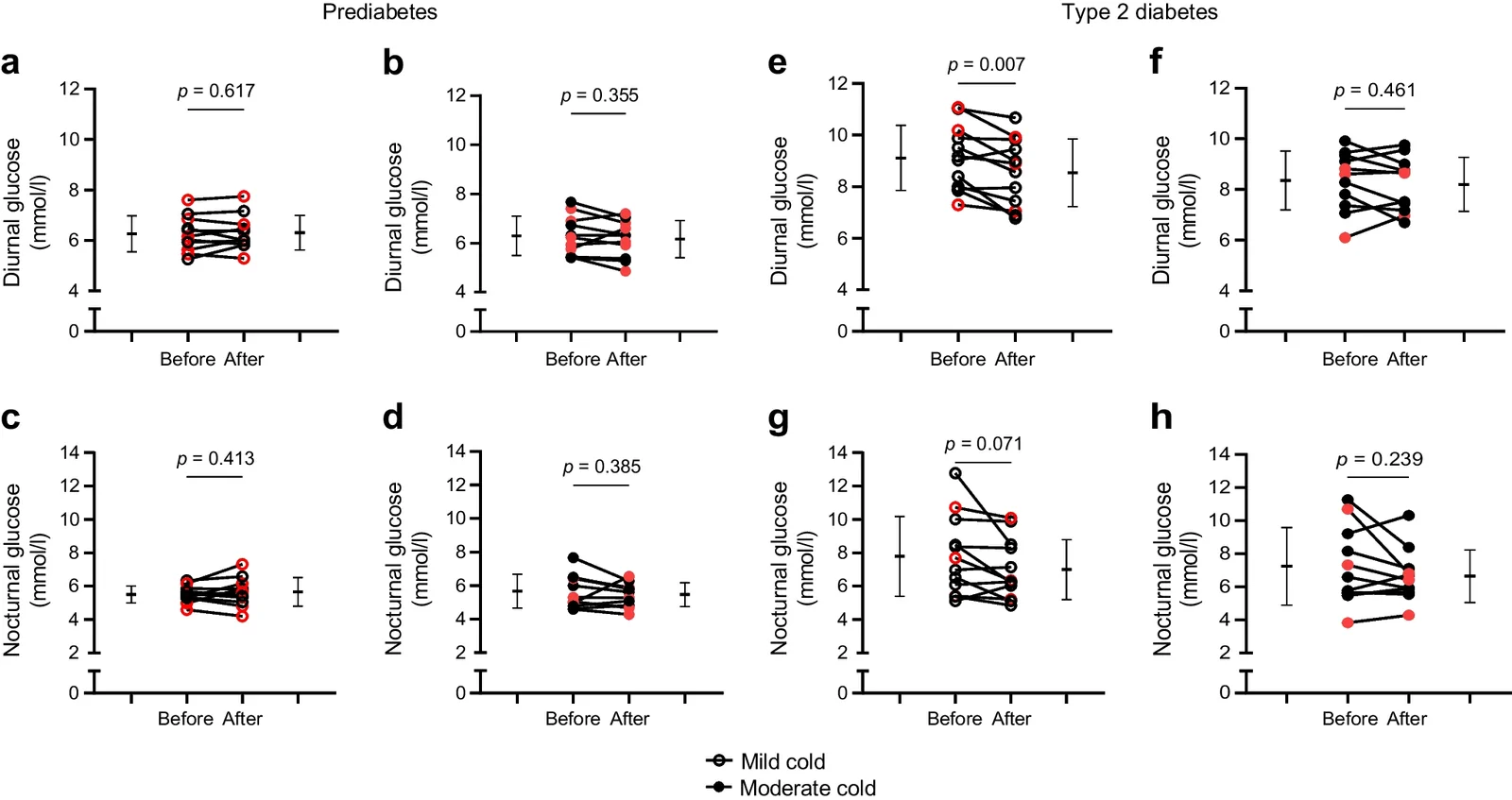
Mean diurnal (**a**, **b**, **e**, **f**) and nocturnal (**c**, **d**, **g**, **h**) interstitial glucose concentration measured before and after mild and moderate cold exposure sessions for the prediabetes (**a**–**d**) and type 2 diabetes (**e**–**h**) groups. All data were analysed with paired *t* test or Wilcoxon signed rank test. Group means are presented as mean ± SD and individual data points are plotted for men (black dots) and women (red dots). Statistical significance was deemed as *p*<0.05

**Fig. 8:**
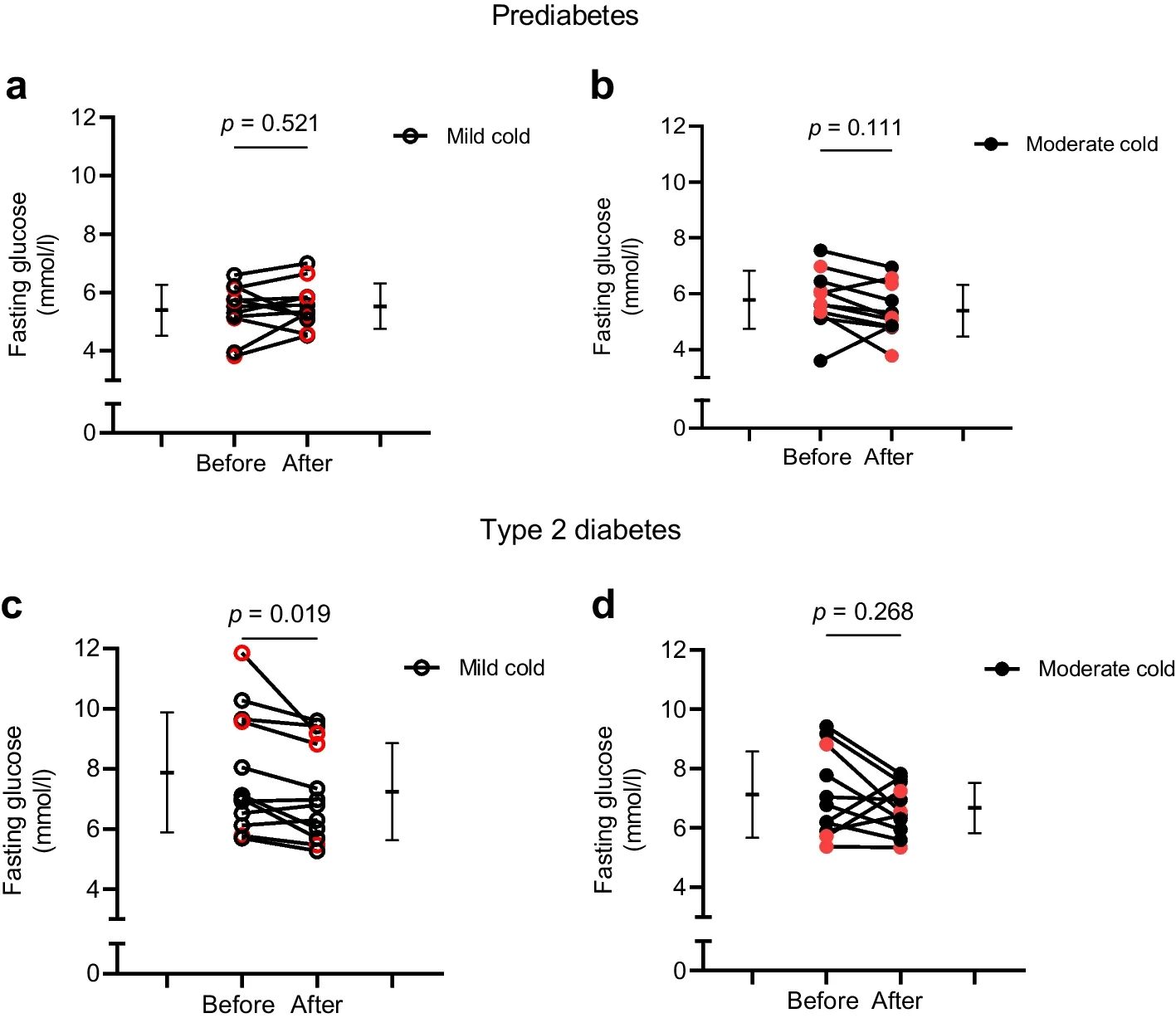
Fasting glucose concentration (mmol/l) before and after the respective cold exposure protocol in prediabetes (**a**, **b**) and type 2 diabetes (**c**, **d**). Group means are presented as mean ± SD and individual data points are plotted for men (black dots) and women (red dots). All data were analysed with paired *t* test or Wilcoxon signed rank test. Statistical significance was deemed as *p*<0.05

In prediabetes, mean lunch-to-lunch interstitial glucose concentration (hereinafter referred to as ‘mean 24 h glucose’) was not different before vs after cold exposure in the mild (*p*=0.426, Fig. 6a) and in the moderate (*p*=0.280, Fig. 6b) cold exposure arms. In type 2 diabetes, mild cold exposure significantly reduced mean glucose levels (−0.6 ± 0.5 mmol/l, *p*=0.003, Fig. 6d). Exposure to moderate cold did not affect glucose levels (*p*=0.168, Fig. 6e). Baseline levels in mild vs moderate cold were not statistically different (*p*=0.338). In prediabetes*,* cold exposure did not affect diurnal, nocturnal or fasting glucose following mild or moderate cold exposure (*p*>0.05) (Fig. 7). In type 2 diabetes, diurnal glucose was significantly reduced after mild (*p*=0.007) but not after moderate cold exposure (*p*=0.461) (Fig. 7e, f). Mean nocturnal glucose also tended to be reduced following mild (*p*=0.071) but not moderate cold exposure (*p*=0.239) (Fig. 7g, h). In type 2 diabetes*,* fasting glucose was also lower following mild (−0.6 ± 0.8 mmol/l, *p*=0.019) but not moderate cold exposure (*p*=0.268) (Fig. 8c, d).

**Fig. 9:**
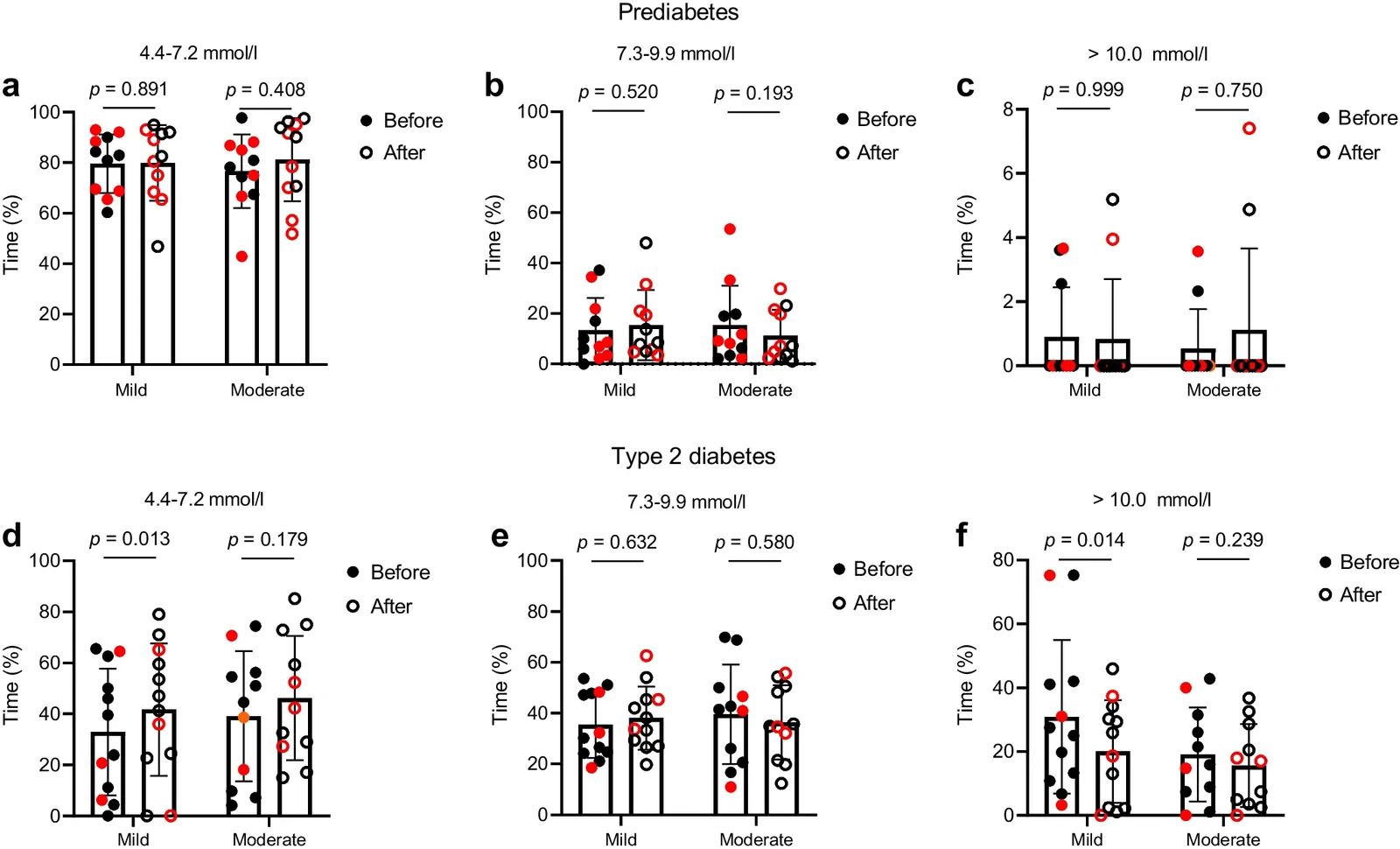
Time spent in normal glucose (4.4–7.2 mmol/l), high glucose (7.3–9.9 mmol/l) and hyperglycaemia (>10.0 mmol/l) before and after mild/moderate cold exposure, expressed as a percentage of the total meal-to-meal time window. Individual data points for the prediabetes (**a**–**c**) and type 2 diabetes (**d**–**f**) groups were plotted, whereby black and red dots represent men and women, respectively. Group means are presented as mean ± SD. All data were analysed with paired *t* test or Wilcoxon signed rank test. Statistical significance was deemed as *p*<0.05

Next, we assessed the time spent in three predefined glucose ranges before and after mild vs moderate cold exposure. In type 2 diabetes, mild cold exposure led to a significant reduction in the time spent in hyperglycaemia (–10.9 ± 12.9%, *p*=0.014) and an increase in the time spent in the normal glucose range (+8.8 ± 10.3%, *p*=0.013). Moderate cold exposure did not affect the time spent in any of the glucose ranges (Fig. 9d–f).

### High fasting glucose at baseline predicts cold-induced reduction in 24 h glucose levels

**Fig. 10:**
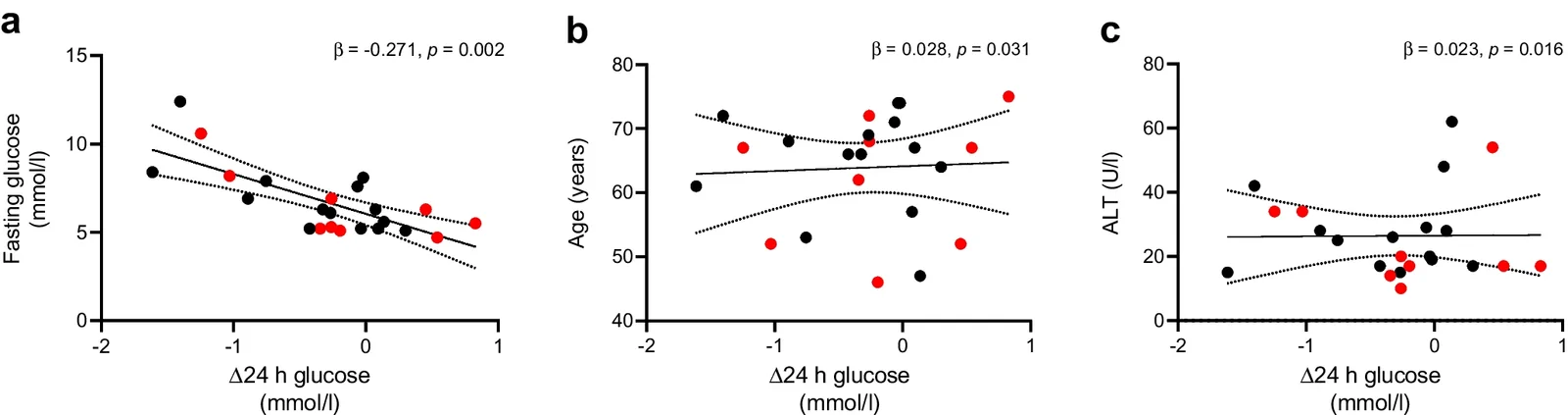
Association of change in 24 h glucose levels following mild cold exposure (Δ24 h glucose) with fasting glucose concentration at baseline (**a**), age (**b**) and fasting ALT levels at baseline (**c**). Associations were identified via multiple linear regression analyses of the pooled group data, whereby black and red dots represent men and women, respectively. Statistical significance was deemed as *p*<0.05

Given the reduction in the 24 h glucose levels, we next explored whether participant characteristics were associated with the response to mild cold. We performed a multiple linear regression pooling the two study groups, with Δ24 h glucose as the dependent variable (ESM Table 5). The best-fitting model included diabetes status, age, baseline FM (kg), FFM (kg), fasting glucose (mmol/l) and ALT (U/l), significantly predicting change in mean 24 h glucose (*p*=0.002, adjusted *R*^2^=0.61). Fasting glucose was a significant predictor (β=−0.271, 95% CI [−0.423, −0.120], *p*=0.002), whereby higher plasma glucose level at baseline was associated with larger reduction in mean glucose (Fig. 10a). Age (β=0.028, 95% CI [0.003, 0.054], *p*=0.031) and fasting ALT at baseline (β=0.023, 95% CI [0.005, 0.041], *p*=0.016) were also significant predictors (Fig. 10b, c). No significant associations were observed between total-body muscle activity (maximum voluntary contraction, MVC [with units %MVC]) and 24 h glucose outcomes (ESM Table 6).

## Discussion

This study examined the acute effects of cold exposure with shivering on 24 h glucose profiles in individuals with type 2 diabetes and prediabetes under mild (1.5 × RMR) and moderate (2.5 × RMR) cold stress. In prediabetes, neither cold intensity altered 24 h glucose profiles. Individuals with type 2 diabetes exhibited a significant reduction in mean 24 h glucose following mild cold exposure, accompanied by lower fasting glucose the next morning, increased time in the normal glucose range and reduced time in hyperglycaemia. These effects were not observed following moderate cold.

Given the beneficial effects of mild cold observed here in individuals with type 2 diabetes, who present with higher fasting glucose levels, our results indicate that (acute) cold exposure may be specifically relevant for those with elevated glucose levels. Correlation analysis supported this, identifying fasting glucose levels as a major determinant of the response of 24 h glucose to mild cold. However, the present study was not powered to detect inter-group differences in response to the cold interventions, and, hence, a firm conclusion about a differential response between prediabetes vs type 2 diabetes cannot be made. Moderate cold did not reduce 24 h glucose levels, even in individuals with type 2 diabetes, despite increasing metabolic rate. Although baseline glucose did not differ significantly between conditions, pre-intervention levels were higher during mild cold, allowing greater potential for reduction. Moderate cold also produced greater variability in physiological parameters, with larger SDs during and after exposure, which may have obscured a statistically significant effect comparable in magnitude to that observed with mild cold.

We also cannot exclude the possibility that moderate cold elicited a more pronounced counterregulatory response. Higher hepatic glucose output during moderate cold may have masked increases in skeletal muscle glucose clearance [21], consistent with evidence that stronger cold stimuli are associated with higher EGP [22], potentially triggered directly by noradrenaline (norepinephrine)-induced increase in glucagon [23], or indirectly by reduced plasma glucose levels during cold exposure [5, 24]. In support of this notion, we observed higher total shivering intensity during moderate vs mild cold in the type 2 diabetes group, suggesting that a certain level of shivering/muscle activity may be needed to induce the counterregulatory responses. Finally, differences in peripheral blood flow during mild vs moderate cold exposure, as evidenced by differences in the reduction in skin temperature between the two intensities, may have differentially affected glucose transport to peripheral tissues during and (immediately) following cold exposure.

Both cold intensities substantially increased EE and fat and CHO oxidation, with larger increases during moderate cold. Notably, due to the individualised protocol, moderate cold exposure duration was longer than mild, and hence the confounding effect of time is inevitable and results should be interpreted in light of this. Plasma glucose levels concurrently decreased during both cold intensities, with larger reductions in type 2 diabetes. Although mechanisms cannot be pinpointed, these may involve increased skeletal muscle glucose uptake, as shown previously in young healthy individuals [6, 7], and/or changes in EGP [22], as well as (brown) adipose tissue (BAT) glucose uptake [25–29]. However, the quantitative contribution of BAT does appear minimal as prospective studies with direct measures of BAT oxidative metabolism and glucose uptake demonstrated that it accounts for only ~1% of whole-body heat production and systemic clearance of glucose [30, 31]. Further, recent evidence from Dumont et al [7] indicates that the rate of oxidative metabolism and glucose uptake in BAT does not differ based on the magnitude of cold exposure and therefore its relative contribution to whole-body metabolism would only decrease with more severe cold.

Elevated NEFA and glycerol levels during both cold exposures and in both groups confirmed enhanced lipolysis, probably sympathetically driven. During mild cold, changes in the two metabolites correlated, but not during moderate cold, which may indicate that higher cold intensities promote greater peripheral NEFA uptake, oxidation and/or re-esterification [7, 32, 33]. The concurrent rise in serum TG suggests increased hepatic output of TG-rich particles and/or reduced peripheral clearance, although the metabolic fate of these particles during cold exposure remains debated [7, 8, 34–36]. Nonetheless, enhanced TG mobilisation is consistent with our previous findings of reduced fasting TG after 10 days of cold acclimation with shivering in individuals with overweight/obesity [4].

From a clinical standpoint, our findings reveal meaningful metabolic benefits for individuals with type 2 diabetes. We observed a 0.6 mmol reduction in mean 24 h glucose, comparable to or exceeding reductions reported after acute aerobic [37–39] and resistance exercise [37], both key components of type 2 diabetes management [40, 41]. Following mild cold exposure, participants with type 2 diabetes also spent ~26% more time in the normal glucose range and ~35% less time in hyperglycaemia, effects again similar to those seen after mild-to-moderate intensity exercise (35–50% *W*_max_) [37–41]. Notably, the observed effects were achieved with approximately half the EE reported for exercise in prior studies [37–39] (~800 kJ during the mild cold exposure vs ~1600–2500 kJ during acute moderate-to-high intensity aerobic exercise), suggesting distinct underlying mechanisms. Evidence indicates that these differences may reflect divergent fuel selection at comparable metabolic rates, with cold exposure eliciting greater recruitment of type II muscle fibres than exercise [10, 42]. Differences in other mechanisms affecting glucose delivery, uptake and phosphorylation in skeletal muscle may also play a role [43]. Our results also suggest that older age may blunt the potential cold-induced reductions in glucose, potentially linked to blunted thermoregulatory responses [44, 45], particularly in older men [46]. However, our study was not sufficiently powered to study sex differences and further research with larger sample sizes and balanced distribution of male and female participants is warranted to explore the relationship between age and sex and cold-induced changes in glucose levels.

To our knowledge, this is the first study to examine 24 h glucose dynamics following acute mild and moderate cold exposure with shivering in individuals with, or at risk for, type 2 diabetes. Strengths include the within-subject design and the robust standardisation of diet and activity. However, the absence of mechanistic measurements prevents identification of the drivers of glucose changes, and future work investigating substrate metabolism and organ-specific glucose handling during and after cold exposure in type 2 diabetes may clarify our observations. Although both sexes were included, the sample size was too small to detect sex differences, despite evidence of sex-specific shivering patterns that may influence substrate use [7, 9]. Follow-up studies should address potential sex-specific effects of cold exposure on glucose homeostasis.

### Conclusion

In conclusion, acute mild cold exposure with shivering lowered mean 24 h and fasting glucose in individuals with type 2 diabetes, improved the time spent in range and reduced the time spent in hyperglycaemia. Participants with prediabetes did not show any changes. The 24 h glucose response to mild cold appeared to be driven by baseline glycaemic status rather than acute substrate partitioning during cold. These findings highlight cold exposure as a promising non-pharmacological adjunct for type 2 diabetes management. Future work should determine the optimal intensity and duration of cold exposure needed to maximise benefits for glucose homeostasis and clarify its clinical utility.

## Supplementary Information

Below is the link to the electronic supplementary material.

ESM1 (PDF 440 KB)

## Data Availability

The current study was registered at ClinicalTrials.gov on 12 December 2022 (registration no. NCT05576025; URL: https://clinicaltrials.gov/study/NCT05576025). Information regarding the trial protocol, statistical analysis plan, randomisation list, source data and any other study materials can be accessed upon request to the corresponding author.

## Abbreviations

BAT: Brown adipose tissue
BMI: Body mass index
CGM: Continuous glucose monitor
CHO: Carbohydrate
EE: Energy expenditure
EMG: Electromyography
FFM: Fat-free mass
FM: Fat mass
LCS: Liquid-conditioned suit
MVC: Maximum voluntary contraction
NEFA: Non-esterified fatty acid
OGIS: Oral glucose insulin sensitivity
OGTT: Oral glucose tolerance test
PAL: Physical activity level
RMR: Resting metabolic rate
TG: Triglyceride
