# Supplementary material for: Acute mild cold exposure with shivering reduces 24 h glucose levels in individuals with type 2 diabetes but not prediabetes
Source: Diabetologia. 2026 Jul 20;69(10):2922–37. doi: 10.1007/s00125-026-06809-z (PMC13541958; doi:10.1007/s00125-026-06809-z)
Supplement: Supplementary file 1 [file 125_2026_6809_MOESM1_ESM.pdf]

Electronic supplementary material

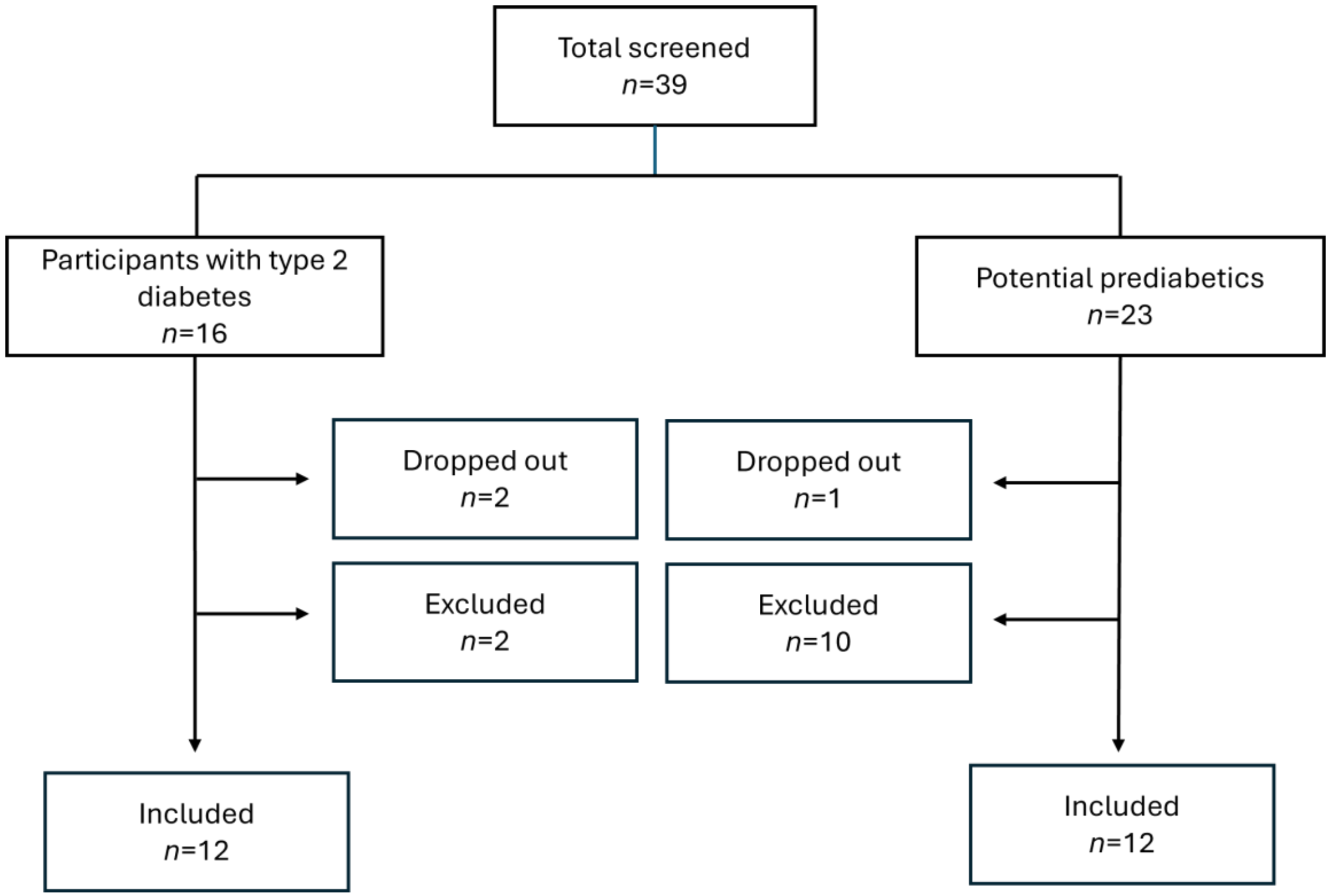

ESM Figure 1. Inclusion chart.

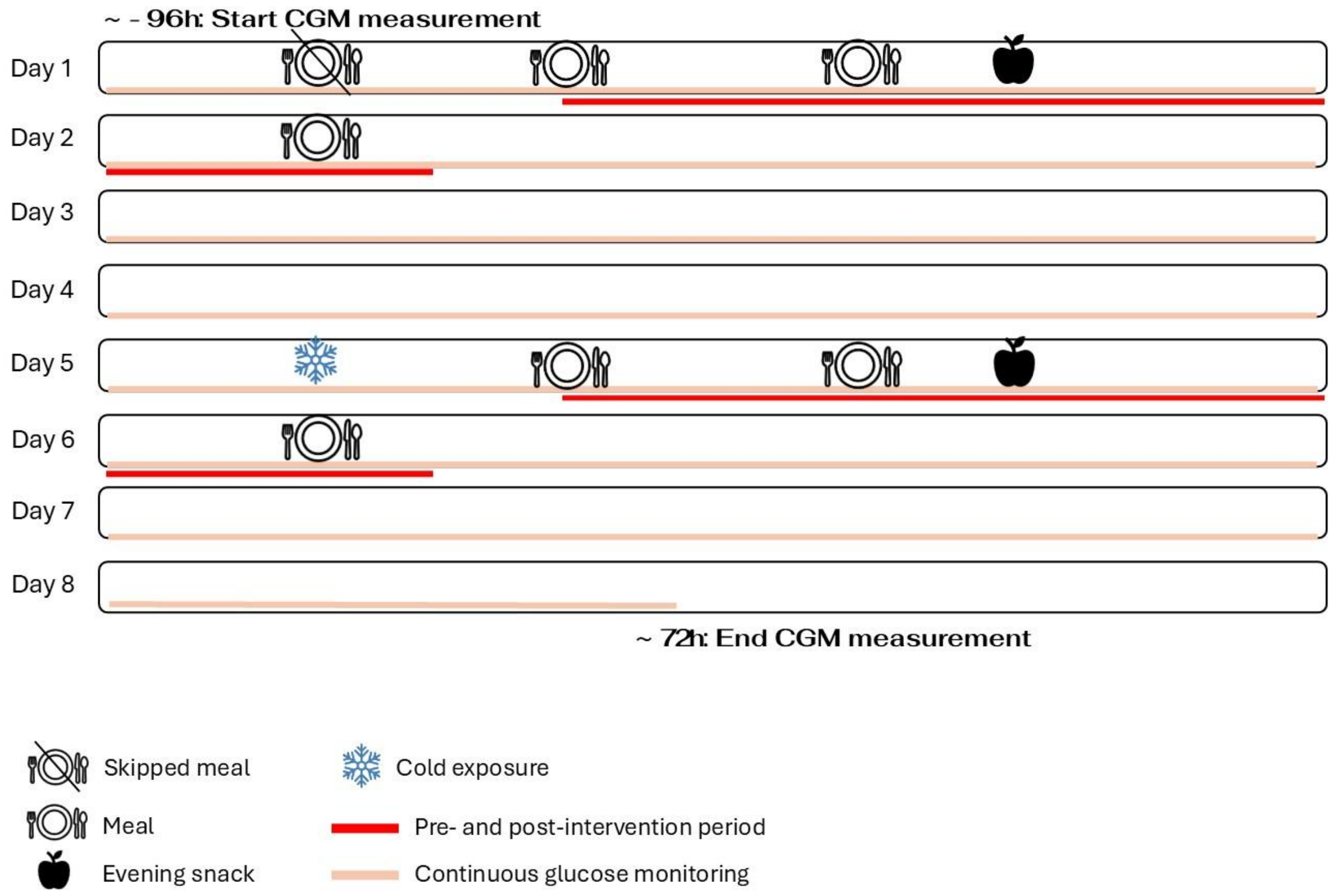

ESM Figure 2. Overview of one study period. Study period begins approximately 96 hours before the respective cold exposure session (at ~09:00). On day 1 subject are instructed to skip breakfast to replicate the cold exposure day and are provided standardised lunch, dinner, evening snack and breakfast the following morning. The pre-intervention time window begins with lunch on day 1 and ends 2 hours following breakfast on day 2. Participants are exposed to cold in the morning of Day 5 (in the fasted state). One hour after cold exposure participants are provided standardised lunch. Post-intervention time window starts with lunch after cold exposure and ends 2 hours following breakfast on day 6.

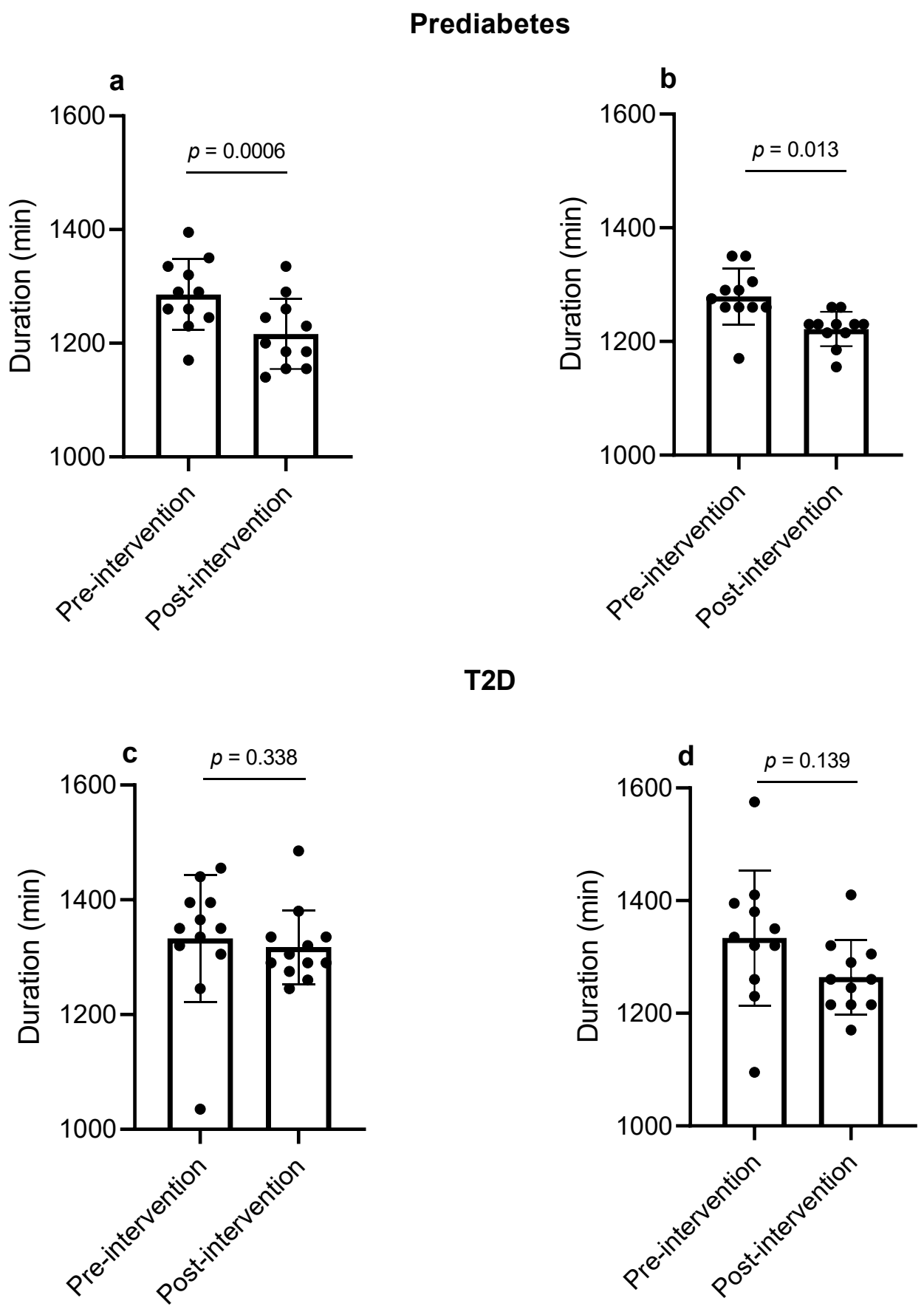

ESM Figure 3. Duration of the pre- and post-intervention meal-to-meal time windows in *Prediabetes* group (a: 'Mild' period and b: 'Moderate' period) and *type 2 diabetes* group (c: 'Mild' period and d: 'Moderate' period).

ESM Table 1. Overview of medications used by participants. Other medications include medications in the following categories: antihistamine (x1), methylfenidaat (x1), osteopenia (x1), blood thinners (x1), prostate (x1), corticosteroid (x1), gastrointestinal (x1), thyroid-regulating (x1), serotonin-reuptake inhibitor (x3).

		**Prediabetes group (*n*=12)**	**Type 2 diabetes group (*n*=12)**
Glucose lowering treatment	Metformin only	NA	*n*=6
	Metformin + sulfonylurea agent and/or GLP-1 agonist	NA	*n*=4
	Diet only	NA	*n*=2
Antihypertensive medications		*n*=2	*n*=8
Cholesterol-lowering medications		*n*=1	*n*=7
Other medications		*n*=7	*n*=7

ESM Table 2. Caloric intake over the ±24 h meal-to-meal period before and after moderate and mild cold exposure for the respective study group. Values are expressed in MJ and % for total intake and macronutrient composition, respectively. Differences are assessed with a paired t-test or non-parametric equivalent. Data are presented as mean ±SD. Statistical significance was deemed as $p<0.05$.

	**Mild cold**			**Moderate cold**		
**Prediabetes group**	**Pre**	**Post**	***p***	**Pre**	**Post**	***p***
Total intake (MJ)	11.9 ± 3.2	12.2 ± 3.2	0.117	11.8 ± 3.2	12.0 ± 3.2	0.282
Fat (%)	32.6 ± 1.4	32.5 ± 1.2	0.506	32.6 ± 1.3	32.4 ± 0.9	0.661
Carbohydrate (%)	49.1 ± 1.6	49.08 ± 1.4	0.901	49.1 ± 1.5	49.2 ± 1.2	0.610
Protein (%)	14.1 ± 0.4	14.4 ± 0.5	0.976	14.4 ± 0.5	14.4 ± 0.5	0.809
**Type 2 diabetes group**	**Pre**	**Post**	***p***	**Pre**	**Post**	***p***
Total intake (MJ)	10.6 ± 2.6	10.6 ± 2.3	0.974	11.0 ± 2.3	10.5 ± 3.0	0.436
Fat (%)	33.9 ± 2.5	34.3 ± 2.3	0.535	34.2 ± 1.7	33.4 ± 3.0	0.283
Carbohydrate (%)	46.5 ± 5.4	47.8 ± 2.2	0.625	46.8 ± 4.6	48.3 ± 2.9	0.625
Protein (%)	15.0 ± 2.0	14.4 ± 1.0	0.221	15.0 ± 1.8	14.6 ± 1.1	0.846

ESM Table 3. Overview of physical activity levels over the ±24-hour meal-to-meal period before and after moderate and mild cold exposure in the respective study group. Differences are assessed with a paired t-test or non-parametric equivalent. Data are presented as mean ± SD. Statistical significance deemed as *p*<0.05.

	**Mild cold**			**Moderate cold**		
**Prediabetes group**	**Pre**	**Post**	***p***	**Pre**	**Post**	***p***
Time sitting/lying (h)	18.3 ± 0.4	17.8 ± 0.5	0.444	18.1 ± 0.3	17.5 ± 0.3	0.085
Time standing (h)	3.2 ± 0.2	2.7 ± 0.2	0.083	3.2 ± 0.3	2.6 ± 0.2	0.180
Time stepping (h)	1.1 ± 0.2	0.9 ± 0.1	0.338	1.0 ± 0.1	0.9 ± 0.1	0.424
**Type 2 diabetes group**	**Pre**	**Post**	***p***	**Pre**	**Post**	***p***
Time sitting/lying (h)	19.5 ± 0.8	18.9 ± 0.6	0.202	18.9 ± 0.7	17.7 ± 0.6	0.055
Time standing (h)	2.4 ± 0.4	2.7 ± 0.4	0.578	2.9 ± 0.4	2.9 ± 0.5	0.924
Time stepping (h)	1.4 ± 0.2	1.6 ± 0.2	0.540	1.3 ± 0.1	1.4 ± 0.2	0.783

ESM Table 4. Plasma concentration of substrates at baseline, at the onset of final 1 hour of cold exposure (*T*=0) and the end of final 1-hour of cold exposure (*T*=60). Data are presented as mean ± SD. Repeated measured AVONA or its non-parametric equivalent was used to test for change over time (time effect presented in the last column for each substrate), with a post-hoc test with Bonferroni or Dunn's correction for multiple testing to determine differences between time points. Statistical differences were denoted as *p<0.05, **p<0.01, ***p<0.001 vs. respective baseline.

		Group *Prediabetes*				Group *T2D*			
		**Baseline**	***T* = 0**	***T* = 60**	***p***	**Baseline**	***T* = 0**	***T* = 60**	***p***
**Glucose (mmol/l)**	Mild	5.8 ± 0.5	5.5 ± 0.3	5.3 ± 0.2*	0.03	8.2 ± 2.0	7.4 ± 1.5	7.0 ± 1.2***	<0.001
	Moderate	5.7 ± 0.5	5.3 ± 0.3	5.3 ± 0.3*	0.02	8.2 ± 1.6	7.0 ± 1.1***	6.7 ± 0.8***	<0.001
**Insulin (pmol/l)**	Mild	123.6 ± 111.7	88.8 ± 72.1	79.0 ± 63.7*	0.02	75.5 ± 43.2	61.8 ± 47.2	46.1 ± 34.3**	0.003
	Moderate	96.5 ± 98.4	76.3 ± 73.2	68.3 ± 69.9**	0.006	88.1 ± 60.4	51.2 ± 27.2*	45.9 ± 34.5*	0.01
**NEFA (mmol/l)**	Mild	0.470 ± 0.139	0.555 ± 0.196	0.658 ± 0.160**	0.001	0.486 ± 0.141	0.543 ± 0.204	0.625 ± 0.217**	0.002
	Moderate	0.448 ± 0.076	0.627 ± 0.146***	0.734 ± 0.140***	<0.001	0.452 ± 0.164	0.520 ± 0.155	0.683 ± 0.230**	0.001
**TG (mmol/l)**	Mild	1.9 ± 0.4	2.3 ± 0.4***	2.4 ± 0.4***	<0.001	1.8 ± 0.9	2.1 ± 1.1*	2.2 ± 1.2***	<0.001
	Moderate	1.7 ± 0.6	2.2 ± 0.7***	2.2 ± 0.7***	<0.001	1.6 ± 0.5	2.0 ± 0.7**	2.1 ± 0.7***	<0.001
**Glycerol (µmol/l)**	Mild	101.3 ± 35.1	130.5 ± 69.5	150.0 ± 52.8*	0.01	65.1 ± 17.0	93.6 ± 31.9**	107.8 ± 38.0****	0.001
	Moderate	75.7 ± 14.5	146.7 ± 59.8	192.7 ± 74.7***	<0.001	57.3 ± 13.5	105.0 ± 39.2**	128.9 ± 53.8****	<0.001

ESM Table 5. Multiple linear regression analyses outcome.

**Variable**	**Estimate**	**Standard error**	**95% Confidence Interval**	***p***
β1: Being a T2D patient	0.156	±0.281	-0.443 to 0.754	0.58
β2: Age (years)	0.028	±0.012	0.003 to 0.054	0.03
β3: Fat mass (kg)	-0.005	±0.010	-0.027 to 0.016	0.60
β4: Fat free mass (kg)	-0.005	±0.010	-0.026 to 0.016	0.61
β5: Fasting glucose (mmol/L)	-0.271	±0.071	-0.423 to -0.120	0.002
β6: ALT (U/L)	0.023	±0.009	0.005 to 0.041	0.02

ESM Table 6. Pairwise correlations between total body shivering activity (% maximum voluntary contraction [%MVC]) and changes in 24 h plasma glucose parameters (Δ = post–pre). Total body average %MVC represents the average across five assessed muscle groups. Lower Δ values indicate greater reductions in glucose parameters. Associations were tested using Pearson or Spearman correlations based on data normality (Shapiro–Wilk test), and corrected for multiple testing via the Hold-Sidak method. Statistical significance was set at *p*<0.05.

	**Total body MVC% (mild cold)**			**Total body MVC% (moderate cold)**			**Total body MVC% (mild and moderate cold combined)**		
	**r**	**95% CI**	***p***	**r**	**95% CI**	***p***	**r**	**95% CI**	***p***
**Δ 24-hour Glucose (mmol/L)**	0.191	-0.241 to 0.559	0.945	0.237	-0.230 to 0.615	0.848	0.180	-0.127 to 0.456	0.761
**Δ Fasting Glucose (mmol/L)**	-0.056	-0.458 to 0.364	0.996	-0.191	-0.584 to 0.275	0.848	-0.168	-0.446 to 0.139	0.761
**Δ Diurnal Glucose (mmol/L)**	0.092	-0.333 to 0.486	0.996	0.475	0.041 to 0.758	0.215	0.284	-0.018 to 0.538	0.375
**Δ Nocturnal Glucose (mmol/L)**	0.068	-0.365 to 0.477	0.996	-0.216	-0.606 to 0.269	0.848	-0.087	-0.386 to 0.228	0.823
**Δ Time in Range (4.4-7.2 mmol/L)**	-0.096	-0.489 to 0.329	0.996	-0.024	-0.462 to 0.423	0.921	0.013	-0.288 to 0.312	0.932
**Δ Time in High Glucose (7.3-9.9 mmol/L)**	0.210	-0.234 to 0.581	0.944	-0.116	-0.531 to 0.344	0.860	-0.128	-0.413 to 0.179	0.798
**Δ Time in Hyperglycaemia (>10.0 mmol/L)**	-0.062	-0.473 to 0.371	0.996	0.445	-0.011 to 0.748	0.260	0.215	-0.101 to 0.491	0.666
